# *Trypanosoma cruzi* Epimastigotes Are Able to Store and Mobilize High Amounts of Cholesterol in Reservosome Lipid Inclusions

**DOI:** 10.1371/journal.pone.0022359

**Published:** 2011-07-27

**Authors:** Miria G. Pereira, Ernesto S. Nakayasu, Celso Sant'Anna, Nuccia N. T. De Cicco, Georgia C. Atella, Wanderley de Souza, Igor C. Almeida, Narcisa Cunha-e-Silva

**Affiliations:** 1 Instituto de Biofísica Carlos Chagas Filho, Universidade Federal do Rio de Janeiro, Rio de Janeiro, Brazil; 2 Department of Biological Sciences, University of Texas at El Paso, El Paso, Texas, United States of America; 3 Diretoria de Programas, Instituto Nacional de Metrologia Normatização e Qualidade Industrial (INMETRO), Rio de Janeiro, Brazil; 4 Instituto de Bioquímica Médica, Universidade Federal do Rio de Janeiro, Rio de Janeiro, Brazil; 5 Instituto Nacional de Ciência e Tecnologia em Biologia Estrutural e Bioimagens, Rio de Janeiro, Brazil; University of Georgia, United States of America

## Abstract

**Background:**

Reservosomes are lysosome-related organelles found in *Trypanosoma cruzi* epimastigotes. They represent the last step in epimastigote endocytic route, accumulating a set of proteins and enzymes related to protein digestion and lipid metabolism. The reservosome matrix contains planar membranes, vesicles and lipid inclusions. Some of the latter may assume rectangular or sword-shaped crystalloid forms surrounded by a phospholipid monolayer, resembling the cholesterol crystals in foam cells.

**Methodology/Principal Findings:**

Using Nile Red fluorimetry and fluorescence microscopy, as well as electron microscopy, we have established a direct correlation between serum concentration in culture medium and the presence of crystalloid lipid inclusions. Starting from a reservosome purified fraction, we have developed a fractionation protocol to isolate lipid inclusions. Gas-chromatography mass-spectrometry (GC-MS) analysis revealed that lipid inclusions are composed mainly by cholesterol and cholesterol esters. Moreover, when the parasites with crystalloid lipid-loaded reservosomes were maintained in serum free medium for 48 hours the inclusions disappeared almost completely, including the sword shaped ones.

**Conclusions/Significance:**

Taken together, our results suggest that epimastigote forms of *T. cruzi* store high amounts of neutral lipids from extracellular medium, mostly cholesterol or cholesterol esters inside reservosomes. Interestingly, the parasites are able to disassemble the reservosome cholesterol crystalloid inclusions when submitted to serum starvation.

## Introduction

Intracellular traffic of cholesterol has been widely studied in mammalian cells [1], [2], [3]. Cholesterol represents a key point in cellular physiology, since it is involved in membrane fluidity, regulation of membrane traffic, microdomain signaling events [4] as well as in many pathologies, such as lysosomal storage diseases, hypercholesterolemia and atherosclerosis [5], [6]. Although mammals are capable to synthesize cholesterol, it is mostly obtained from diet. Intracellular acquisition of cholesterol is carried out through low density lipoprotein (LDL) receptor-mediated endocytosis [7]. From the plasma membrane LDL particles are transported to the early endosomal network and move through multivesicular bodies (MVB) in late endosomes/lysosomes (LE/LY) direction [8], [9]. Interestingly, MVB comprise 60% of cholesterol reservoir in the endosomal system [1], [2]. Cholesteryl ester hydrolysis may begin in early endosomes and free cholesterol would be almost completely available in LE/LY [10]. Cholesterol efflux for cellular distribution is guaranteed by the participation of the transmembrane NPC1, soluble NPC2 proteins and by sterol-sensing proteins, although the precise mechanism is not well understood yet [11]. Cholesterol excess is stored as cholesteryl esters in lipid droplets, well characterized in adipocytes and steroidogenic cells [12].

*Trypanosoma cruzi*, the etiologic agent of Chagas disease, does not synthesize cholesterol. Remarkably, the major sterol produced by this protozoan is ergosterol, whose biosynthetic pathway is an important target for chemotherapy [13]. Nonetheless, epimastigotes, the proliferative noninfective forms that live in the insect-vector midgut, acquire cholesterol through the uptake of LDL from the hematophagus insect diet [14]. However, the cellular implications of lipid uptake are unknown. LDL, as all other macromolecules, uptake occurs at two unique membrane invaginations: the cytostome and the flagellar pocket [14], [15]. From both entry sites, cargo-containing vesicles are originated and fuse with early endosomes [15]. Afterwards, the cargo is delivered to the last organelle of *T. cruzi* endocytic pathway, the reservosome (for a recent review see [16]). Reservosomes are lysosome-related organelles [17] responsible for cellular digestion of endocytosed material and for providing substrates to cellular metabolic demands and metacyclogenesis, in a modulated manner [18]. Proteomic analysis of isolated reservosomes and their membranes confirmed the presence of many proteases and identified enzymes associated with lipid metabolism, as well as ABC transporter family members and mammalian Rab 18 homologue, which are involved in lipid transport [19].

Morphologically, reservosomes are rounded organelles (400–600 nm) placed at the posterior region of the parasite [20]. Organelle protein matrix is interposed with internal membranes and unusual lipid inclusions displaying disk or rod-like rectangular shapes [21]. Moreover, reservosome lipid inclusions are surrounded by a phospholipid monolayer, thus resembling the cholesterol crystals present in lysosomes of foam cells, as a result of the intense acquisition of lipoproteins by endocytosis [22], [23]. When cholesterol and cholesteryl ester masses reach a critical point, the crystallization of lipids is favored.

Preliminary biochemical analysis of isolated reservosomes revealed cholesterol (cholesteryl esters) and ergosterol as the major neutral lipids in these organelles [24]. However, the biochemical composition of reservosome lipid inclusions remains unknown, as well as their function in the parasite biology.

In this work we established a protocol for lipid inclusion isolation, starting from a purified reservosome fraction, in order to determine their composition by GC-MS. We showed that rectangular and spherical lipid droplets are formed mostly by cholesterol and their formation is dependent on serum supply. Moreover, we demonstrated that epimastigotes are capable of consuming cholesterol stock in reservosomes, including rod like inclusions, during periods of serum starvation.

## Materials and Methods

### Parasites

*T. cruzi* epimastigotes (Y strain) were cultivated for 4–5 days at 28°C in LIT (liver-infusion tryptose) medium [25], supplemented with 1, 10 or 50% fetal calf serum (FCS) (Vitrocell, São Paulo, Brazil). Alternatively, parasites were grown in 10% delipidated FCS (dFCS) supplemented with different purified LDL concentrations. In order to determine epimastigote proliferation in different serum concentrations, 1×10^6^ epimastigotes per mL were cultivated in LIT supplemented with 1, 10 or 50% of FCS at 28°C for 24, 48, 72 and 96 h. Cellular growth was measured by direct counting in a Neubauer chamber.

### Lipoprotein purification

Low density lipoprotein (LDL) was purified from fresh human plasma as described [26] with some modifications. Briefly, KBr was added to 15.0 mL of human cell-free plasma to adjust the density to 1.3 g/cm^3^. The plasma was added to a vertical angle centrifuge tube with 20.0 mL of saline solution (150 mM NaCl, 1 mM EDTA). This material was ultracentrifuged at 150,000 g in a vertical angle Beckman RPVTi 50 rotor (Beckman Coulter Inc, Fullerton, CA, USA) at 4°C for 18 hours. The LDL fraction was localized and removed. KBr was added again to adjust the density to 1.2 g/cm^3^, and the material was ultracentrifuged at 150,000 g in the same rotor at 4°C for 18 hours. The purified lipoprotein were extensively dialyzed against PBS with 1 mM EDTA. The lipoprotein concentration was quantified using DC Protein Assay (Bio Rad). For the uptake assays and culture, the LDL was sterilized by filtration with a 0.22 µm membrane (Millex-GV, Millipore S.A., Molsheim, France). The integrity of the sterile LDL was verified in a polyacrylamide (3–15%) slab gel under denaturing conditions.

### Fetal calf serum delipidation

Lipid extraction of fetal calf serum (FCS) (Cultilab Ltda., Campinas, SP, Brazil) without protein precipitation was performed as described [27].

### Nile Red staining and fluorimetric analysis

*Trypanosoma cruzi* epimastigotes (5×10^6^ cells) from LIT medium supplemented with 1, 10 or 50% FCS were washed twice in PBS (phosphate-buffered saline: 10 mM sodium phosphate buffer, pH 7.2, plus 150 mM sodium chloride) and fixed in 4% freshly prepared formaldehyde in PBS for 20 min at room temperature. After washing twice in PBS, cells were incubated in 10 µg/mL Nile Red for 15 min at room temperature, protected from light. The parasites were washed in PBS, pH 7.2, and incubated in DAPI (5 µg/mL) for 5 min. The cellular suspensions were transferred to a black 96-well microplate and Nile Red fluorescence was determined in a Microplate Reader Spectra Max M2 (Molecular Devices): yellow-gold fluorescence of neutral lipid inclusions were acquired (excitation: 485 nm; emission 535 nm). An aliquot of each cell suspension was collected and adhered to 0.1% poly-L-lysine coated glass coverslips. Samples were mounted on 0.2 M *n*-propylgallate in glycerol∶PBS (9∶1) and the yellow-gold fluorescence images of neutral lipid inclusions were acquired using appropriated filters in a Zeiss Axioplan epifluorescence microscope coupled to an Olympus X30 CCD camera. Images were further processed using Adobe Photoshop CS2 (Adobe Systems, Inc.). Nile Red had already been used to quantify and image lipid inclusion formation inside acidic organelles [23].

### Reservosome and lipid inclusion isolation

A reservosome purified fraction from parasites cultivated in 10 and 50% FCS medium were obtained as described [19], [24] and resuspended in TMS buffer (20 mM Tris-HCl, pH 7.2, 2 mM MgCl_2_, 250 mM sucrose) supplemented with protease inhibitor cocktail (Sigma-Aldrich P2714). For lipid inclusion isolation, three milliliters of isolated reservosomes (1.5–2 mg/mL) were washed in TMS, pH 7.2, and centrifuged at 120,000×*g* for 15 min at 4°C in a Beckman SW28 centrifuge tube. The pellet was resuspended in 3 mL 200 mM Na_2_CO_3_, pH 11.5, at room temperature for 15 min. The sample was then sonicated at 10% of total amplitude (Sigma, GEX 600 Model) using a standard probe (13 mm radiating diameter), for three cycles of 10 s, with 5-s rest between cycles. Sample volume was completed to 7 mL with the alkaline solution and the suspension was transferred to a SW 40 centrifuge tube. Sample was overlaid with 3.5 mL 20 mM Tris-HCl, pH 7.5, containing 100 mM KCl and 2 mM MgCl_2_, and the tube was centrifuged at 274.000×*g* for 1 h a 4°C. A white band was collected at the top of the gradient, mixed with 3 mL TMS, pH 7.5, and deposited at the bottom of a Beckman SW50.1 centrifuge tube, overlaid with additional 2 mL 20 mM Tris-HCl, pH 7.5, 100 mM KCl and 2 mM MgCl_2_, and centrifuged at the same conditions described before. The white band was recovered and processed for electron microscopy or frozen at −20°C to subsequent biochemical analysis.

Alternatively, reservosome fraction was adhered to 0.1% poly-L-lysine coated glass coverslips for 20 min and fixed in 4% formaldehyde in PBS for 10 min at room temperature. The samples were washed in PBS, followed by Nile Red incubation (10 µg/mL in PBS) for 15 min, protected from light. Finally, reservosomes were washed in PBS and Nile Red labeled lipid inclusions observed as described before.

To quantify the neutral lipid content of isolated reservosomes from 10 and 50% FCS by fluorimetric analysis, reservosomes isolated from both cultures were washed in PBS and prepared for Nile red fluorimetric analysis as described in the previous section.

### Electron Microscopy

#### a) For ultrathin sections

Epimastigotes (1, 10 and 50% FCS) and isolated reservosomes were fixed in 2.5% glutaraldehyde and 4% formaldehyde in 0.1 M sodium phosphate buffer, pH 7.2, for 60 min at room temperature, postfixed in 1% osmium tetroxide, 0.8% potassium ferrocyanide, 5 mM calcium chloride in 0.1 M cacodylate buffer, pH 7.2, for 60 min, dehydrated in an acetone series and embedded in Epoxi resin. Ultrathin sections were stained with 5% uranyl acetate and lead citrate and observed in a Zeiss 900 transmission electron microscope operating at 80 kV.

#### b) Uranyl Acetate Stained Preparations

Isolated lipid inclusions were visualized under the electron microscope as described [28] with some modifications. The fraction was put onto a Formvar coated nickel grid for 25 min at room temperature, washed by inversion over deionized water and fixed in 2% glutaraldehyde in 0.1 M phosphate buffer, pH 7.6, for 15 min and washed again in water. Subsequently, the fraction was stained in 2% OsO_4_ in 0,1 M sodium phosphate buffer, pH 7.6, for 20 min, washed in water, incubated for 10 min in 4% uranyl acetate and observed in a Zeiss 900 transmission electron microscope operating at 80 kV.

### Morphometric analysis

Morphometric measurements were performed with images acquired at the magnification of 30.000×(1 micrometer corresponds to 533 pixels) at 80 kV using MET ZEISS 900. All cells in each random section were registered until reaching 50 different cells. The areas occupied by reservosomes and by the lipid inclusions inside them were measured and processed using Image J.

### Lipid extraction

Isolated reservosomes and lipid inclusions (in 13×100 mm PYREX clear borosilicate glass tubes with Teflon-lined screw cap) were extracted three times in 10 volumes of chloroform (CHCl_3_)/methanol (CH_3_OH) solutions: CHCl_3_∶CH_3_OH∶H_2_O (1∶2∶0.8, v/v/v) and CHCl_3_∶CH_3_OH (2∶1, v/v) [29]. After adding each solvent solution, the tube was vortexed for approximately 1 min, then centrifuged at room temperature for 30 min at 2000×*g*. Following centrifugation, the organic phase was transferred to a clean glass tube with Teflon-lined cap and stored at −20°C until further use. The sample was dried under highly pure nitrogen stream after the last extraction in each step as described by [30].

Samples were analyzed by one-dimensional thin-layer chromatography (TLC) on Silica Gel 60 plates (E. Merck, Darmstadt, Germany) for neutral lipids using n-hexane∶diethyl ether∶acetic acid (60∶40∶1 v/v). Cholesterol, cholesteryl-oleate, glycerol-tryoleate, diolein, oleoyl-glycerol and oleic acid (Sigma Chemical Co, St Louis, MO, USA) were used as standards. The lipids were visualized using a charring reagent (CuSO_4_) after heating at 200°C for 20 min. After that, the chromatography plates were digitized.

### Sterol purification

Glass columns prepared in Pasteur pipettes were packed with fine glass wool and approximately 100 mg silica gel resin (pore size 60 Å, 70–230 mesh, Sigma Aldrich). After washing with CH_3_OH and acetone columns were equilibrated with CHCl_3_. Following equilibration, the lipid sample dissolved in 1 mL CHCl_3_ was loaded onto the column. Neutral lipids and free fatty acids were eluted out with 2–3 mL CHCl_3_. This was followed by 2–3 mL acetone to elute glycolipids and ceramides, and 2–3 mL methanol to elute phospholipids [31]. All samples were dried under nitrogen stream and stored at −20°C until further use.

### Gas chromatography–mass spectrometry (GC–MS) analysis

The analysis of the sterol fraction by GC-MS was carried out as described [32]. For analysis of fatty acids by GC-MS, total lipids of reservosomes were isolated as described above. Alkaline hydrolysis of total fatty acids was carried out following a method adapted from [33]. Twenty-five-microliter aliquots of the total lipid extracts were dried under nitrogen stream, resuspended in 100 µL 13N ammonium hydroxide: methanol (1∶1, v∶v), incubated for 1 h at 37°C and then dried under nitrogen stream. The samples were washed twice with 100 µL dry methanol, with complete drying under nitrogen stream between each wash. For the methylation, 100 µL 0.5N methanolic HCl (Supelco, Sigma-Aldrich) was added, and the reaction mixture was incubated for 1 h at 75°C. The reaction mixture was allowed to cool to room temperature and then neutralized with 100 µL 0.5 N NaOH. To remove HCl from the reaction, samples were washed each with 1 mL deionized water and dichloromethane (DCM). The aqueous phase was extracted and samples were washed two more times with water. Finally, the organic phase was transferred to a fresh tube and briefly dried under nitrogen stream [33].

For GC-MS analysis, samples were redissolved in 100 µL DCM and 1 µL was used for analysis in a Trace-GC gas chromatographer (Thermo Fisher Scientific, Austin, TX) coupled to a mass spectrometer (Polaris Q, Thermo Fisher Scientific) (GC-MS). Samples were separated in a SP-2380 fused silica column (30 m×250 µm×0.20 µm, Supelco, Sigma-Aldrich). The injector was set at 200°C, and the following gradient was used: 70°C for 5 min, followed by 4°C/min up to 140°C, 2°C/min up to 185°C, and 185°C for 10 min. Helium was used as the carrier gas, with a flow rate of 1 mL/min. The molecules were ionized by electron impact at 70 eV and 200°C. The spectra were collected in the 30–400 *m/z* range, and a chromatogram was generated by plotting the spectra of diagnostic fragment-ion species for *m/z* 41, 43, and 55. Fatty-acid species were identified by comparison with the FAME 37 methylated FA mix standard (Supelco, Sigma-Aldrich).

### Lipid consumption

Aiming to determine the lipid reservoir consumption of epimastigotes from medium supplemented with 10 or 50% FCS, parasites were washed in PBS, transferred to serum-free LIT medium and incubated for 0, 8, 24, 48, or 72 h at 28°C. After each time, 5×10^6^ cells were washed in PBS and incubated in Nile Red (10 µg/mL in PBS) for 15 min in the dark. Then, the parasites were washed in PBS, followed by incubation with 1 µM Sytox Blue (Invitrogen) for 5 min to determine cell viability. For positive control, 5×10^6^ cells were permeabilized for 5 min with 0.1% Triton X-100 in PBS. The quantification of fluorescence was determined as describe before. In addition, parasites were processed for observation by transmission electron microscopy and morphometric analysis as described above.

## Results

### 1. Epimastigote lipid content depends on lipid supply

**Figure 1:**
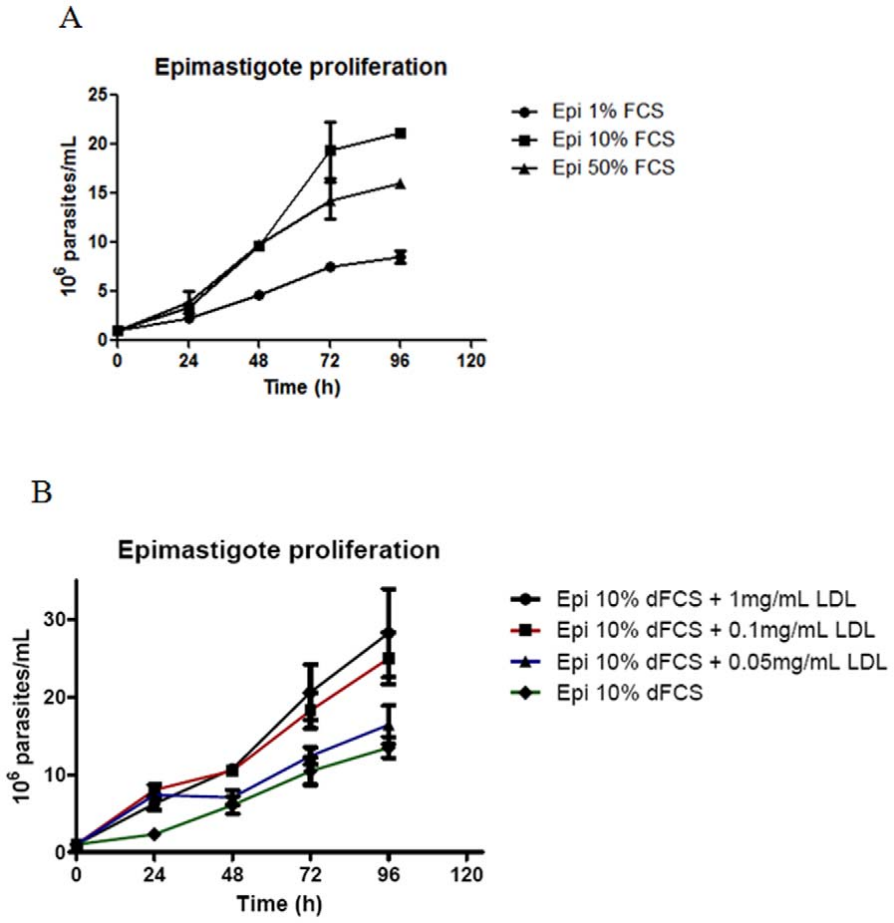
Effect of FCS concentration on the epimastigote growth. Epimastigotes were cultivated as usual (LIT supplemented with 10% FCS) or in LIT supplemented with 1% or 50% FCS (**A**). Alternatively, parasites were cultivated in LIT supplemented with delipidated FCS and growing concentrations of purified LDL (**B**). Each point corresponds to the mean of three independent experiments in quadruplicate. The data was analyzed with Two-way ANOVA test and post analyzed by Bonferroni test (P<0.01).

We decided to modulate FCS concentration in epimastigote studies *in vitro* to verify cell proliferation in low and high concentrations of serum. Statistical analysis showed that cell proliferation is dependent on serum concentration, as observed in Figure 1A. After 24 hours, it is possible to note a significant difference in culture growth (P<0.01). We observed that epimastigotes were capable to divide either in low (1%) or high (50%) serum concentrations, although they were more proliferative in 10% FCS at 96 h of growth. To evaluate the influence of other serum components in parasite proliferation, we cultivated epimastigotes in LIT supplemented with 10% delipidated fetal calf serum (dFCS) and added purified low density lipoprotein. The necessary control of medium delipidation and supplentation with purified LDL was performed by TLC (Figure S1). The result confirmed that parasites survive and proliferate in low LDL concentration as well as in concentrations as high as 1 mg/mL (Figure 1B). Thereafter, we decided to work modulating serum concentration.

**Figure 2:**
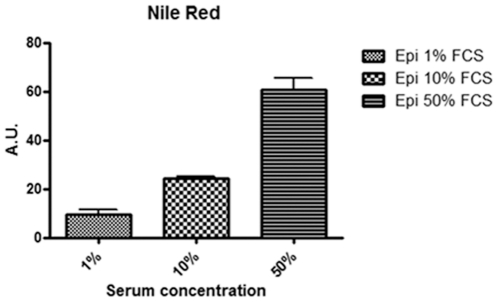
Neutral lipid content as a function of FCS concentration. Fluorimetric analysis using Nile Red reveals that epimastigotes cultivated in 50% FCS store more neutral lipids than those grown in 10 and 1% FCS. Fluorescence intensity was expressed in arbitrary units. The results are from two independent experiments in triplicate. The data was analyzed with One-way ANOVA test (P<0.05).

**Figure 3:**
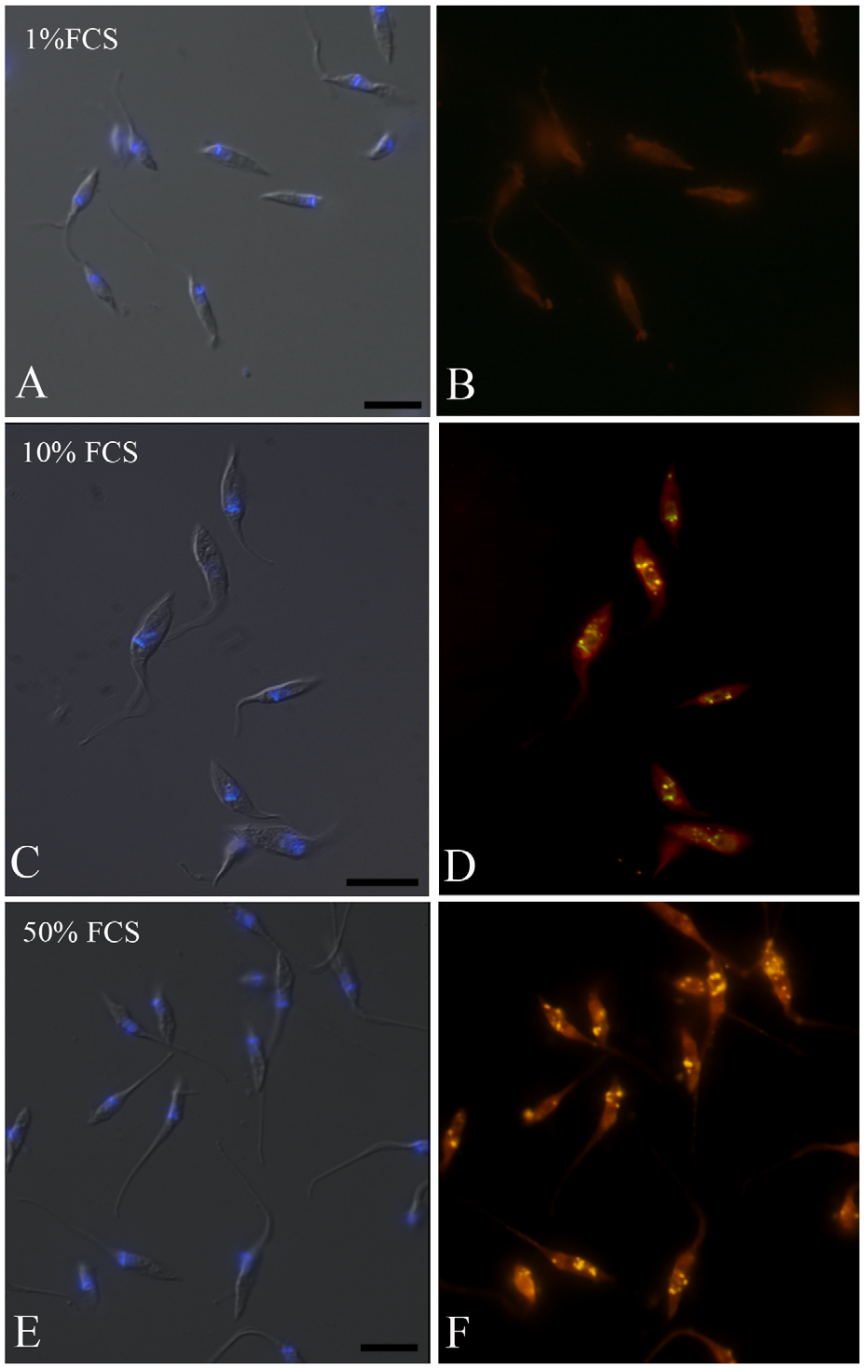
Fluorescence microscopy of epimastigotes cultivated in different FCS concentrations and incubated with Nile Red. (**A**, **C**, **E**) Differential interferential contrast (DIC) and DAPI images of epimastigotes grown in 1%, 10% and 50% FCS, respectively, showing kinetoplast and nucleus position; (**B**) Lack of staining in reservosomes treated with Nile Red. (**D**, **F**) Reservosomes of epimastigotes cultivated in 10% and 50% FCS, respectively, typically localized in the posterior region of the cells and positively stained in yellow with Nile Red. Bars: 10 µm.

**Figure 4:**
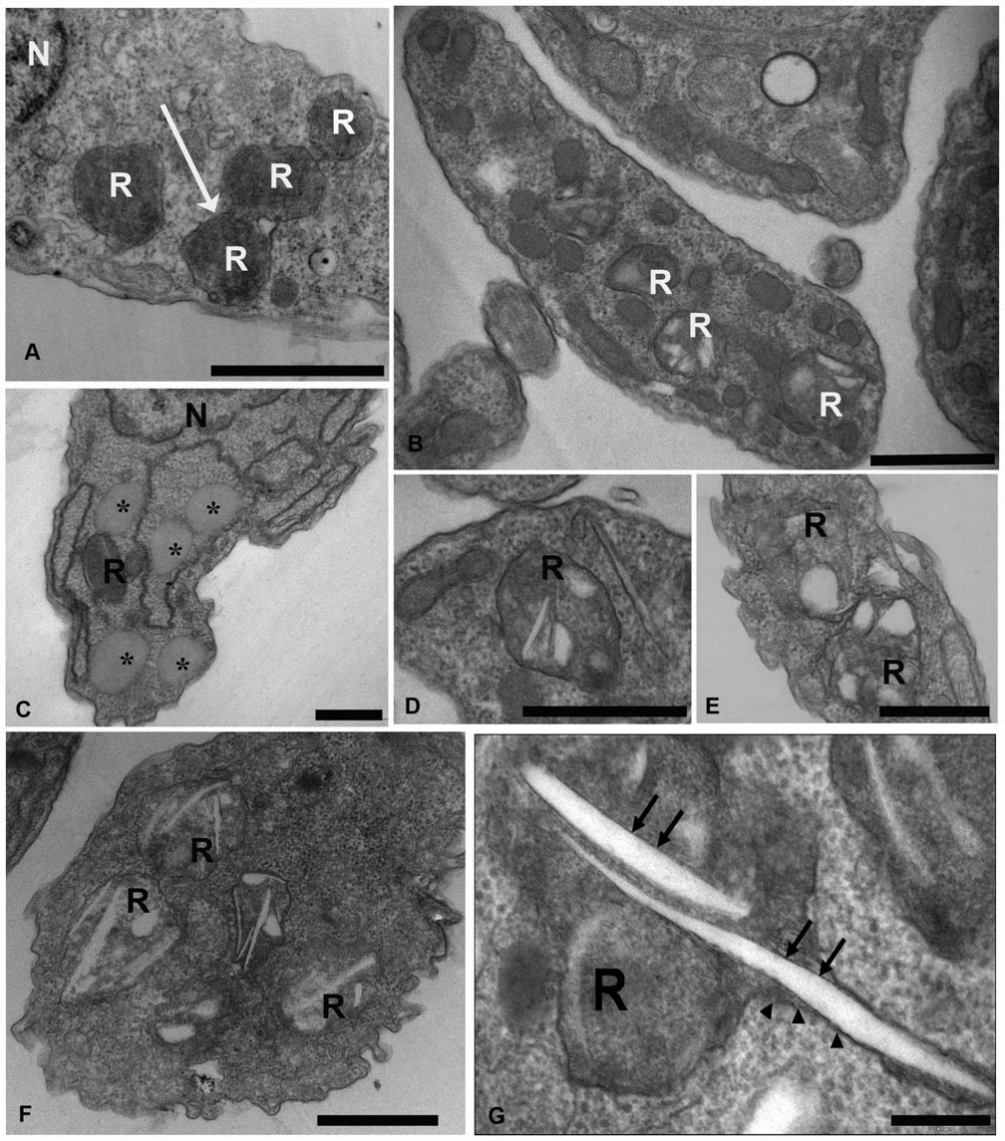
Electron microscopy of epimastigotes cultivated in LIT medium supplemented with 1, 10 or 50% FCS. (**A**) Epimastigotes grown in 1% FCS - Reservosomes at the posterior region of the parasite are devoid of lipid particles. The images suggest fusion events (arrow). (**B**, **C**, **D**) Epimastigotes grown in 10% FCS – reservosomes show many lipid inclusions, some of them having a rectangular shape. Cytoplasmic lipid bodies were also observed (asterisks). (**E**, **F**, **G**) Epimastigotes grown in 50% FCS - Reservosomes loaded with many rectangular inclusions. (G) Sword-shaped lipid inclusions in reservosomes that are crossing the organelles. Note the presence of a phospholipid monolayer (arrows) involving the inclusions, while a phospholipid bilayer surrounds the organelle (arrowheads) R, reservosome; N, nucleus. Bars: 0.5 µm.

**Figure 5:**
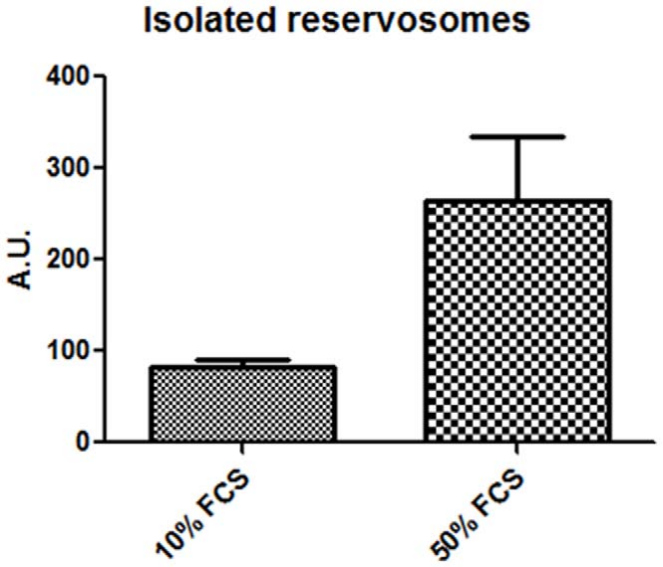
Fluorimetric analysis of isolated reservosomes from epimastigotes maintained in 10 and 50% FCS. Fluorescence intensity was measured by its emission at 535 nm and expressed in arbitrary units. To make Nile Red fluorescence measurements comparable, each sample corresponded to 50 µg of reservosome protein fraction (DC Protein Assay, Bio Rad). Results are from three independent experiments. The data was analyzed with T-test and post analyzed by One-tailed test (P<0.05).

Subsequently, we hypothesize that the epimastigote total lipid content would vary in concentration according to sterol uptake from the parasite diet. To test this hypothesis we collected the parasites from medium with 1, 10 and 50% FCS and incubated cells in Nile Red for neutral lipid fluorescence staining and performed a fluorimetric analysis (Figure 2). We observed that epimastigotes grown in 50% FCS stored more neutral lipids than parasites cultivated in medium supplemented with 10% FCS, as usual, and much more than parasites cultivated with 1% FCS. We have also quantified neutral lipid content of epimastigotes cultivated in LIT with 10% dFCS, supplemented or not with 1 or 2 mg/mL LDL and compared with control parasites cultivated in 10% complete FCS. The results (Figure S2) confirmed the hypothesis that epimastigote neutral lipids come from medium, as those incubated in 10% dFCS for only 48 h hours contain about half the lipid content of control parasites. On the other hand, after staying in 10% dFCS for 48 hours, the incubation with 1 mg/mL LDL for 24 additional hours raised neutral lipid content about 6 times and incubation with 2 mg/mL LDL in the same conditions resulted in neutral lipid concentration 8 times higher than in control parasites. Moreover, the fluorescence microscopy analysis showed a yellow-gold fluorescence concentrated in internal compartments of epimastigotes from 10 and 50% FCS supplemented media (Figures 3C–F), while no internal stocks were detected in 1% FCS epimastigotes (Figures 3A–B). Ultrathin sections showed reservosomes devoid of lipid inclusions from epimastigotes cultivated in 1% FCS (Figures 4A). Images suggestive of reservosome homotypic fusion were frequently observed in these parasites. Epimastigotes cultivated with 10% FCS had both round and rod shaped lipid inclusions (Figures 4B–D). In these parasites we could also observe cytoplasmic lipid bodies (Figure 4C). In comparison, large and lipid-loaded reservosomes from parasites supplemented with 50% of FCS (Figures 4E–G) presented many rod, disc or sword shaped lipid inclusions that could protrude from organelle limits. Additionally, fluorimetric analysis of isolated reservosomes from epimastigotes grown in 50% FCS showed over twice the amount of neutral lipids than reservosomes from parasites cultivated in 10% FCS (Figure 5). Together, these results corroborate the idea that the neutral lipid storing in reservosomes is dependent on serum content uptake by endocytosis.

### 2. Cholesterol is the major neutral lipid in reservosomes

**Figure 6:**
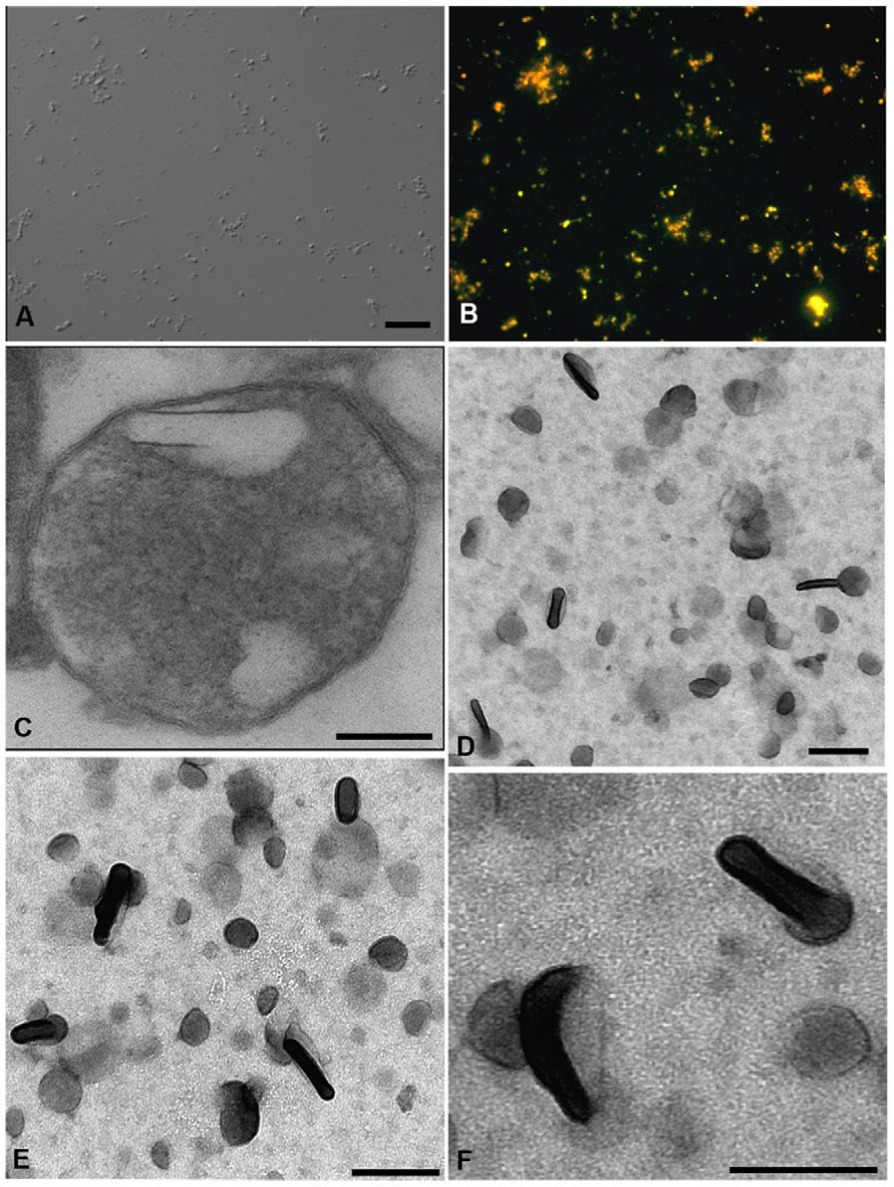
Isolation of reservosome lipid inclusions. (**A**) DIC and (**B**) Nile Red fluorescence of reservosomes isolated from epimastigotes cultivated with 10% of FCS. (**C**) Ultrathin section of an isolated reservosome. (**D**, **E**, **F**) Whole mount osmium tetroxide and uranyl acetate-stained electron microscopy of the reservosome lipid inclusion fraction, showing rectangular and spherical forms. Note the morphological similarity between isolated inclusions and those inside intact reservosomes (C). Bars (A, B) 5 µm, (C) 0.15 µm, (D, E) 0.2 µm, (F) 0.15 µm.

In order to determine the biochemical nature of reservosome lipid inclusions, we had first obtained a purified fraction of isolated reservosomes as previously performed before by our group [19], [24]. Here, we developed a protocol to isolate the internal lipid inclusions starting from the reservosome purified fraction. As expected, the reservosome fraction was positively stained with Nile Red (Figures 6A–B). Moreover, the electron lucent lipid particles were further visualized by ultrathin section inside the reservosome and by whole mount electron microscopy (Figures 6C–F). Based on the microscopy analysis, the predominant structures were rectangular or spherical, like those found inside isolated whole organelles and *in situ*. Thus, our protocol appears to produce an enriched fraction of reservosome lipid inclusions, since no other structures, such as contaminant membranes and organelles, were evident.

**Figure 7:**
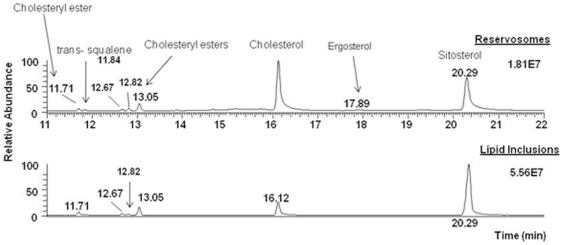
Neutral lipid analysis of reservosome fractions. Lipid inclusions and isolated reservosomes were subjected to lipid extraction with chloroform/methanol and chloroform/methanol/water and fractionated in a Silica 60 column. The sterol-rich fraction was dried under nitrogen stream, resuspended in dichloromethane and analyzed by GC-MS (Trace GC - Polaris Q Thermo Fisher Scientific, column TR5-ms, Thermo). Sitosterol was used as internal standard. Cholesterol (52.9%) and cholesteryl ester (47.1%) are the only neutral lipids detected in isolated reservosome lipid inclusions.

Additionally, when the reservosome lipid fraction was subjected to GC-MS analysis, we found out that the major neutral lipids were cholesterol and cholesteryl esters (Figure 7). No other sterols, like ergosterol or ergosterol ester, were detected. This highlights the hypothesis that reservosome lipid inclusions were a result of lipid endocytosis, storage and lipid crystallization of cholesterol from medium inside the reservosomes. When we compared with reservosome fraction, cholesterol appeared as the major sterol (84.0%) (highest peak area), followed by cholesteryl esters (11.0%), ergosterol (1.5%) and squalene (3.5%) (Figure 7).

**Figure 8:**
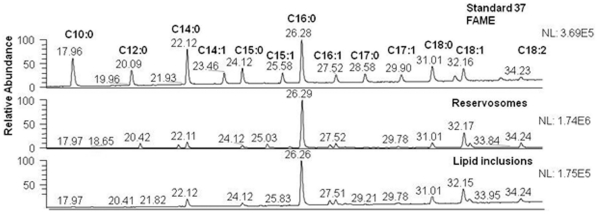
Fatty acid analysis of neutral lipids of the reservosome fraction. Sterol-rich fraction was hydrolyzed with NH_4_OH and methylated with methanolic HCl, extracted in dichloromethane and analyzed by GC-MS, revealing palmitate (C16:0) as the major fatty acid of reservosome fraction.

The more predominant fatty acid species covalently linked to cholesterol both in reservosome and lipid fractions was palmitate (C16:0), followed by oleate (C18:1), stearate (C18:0) and myristate (C14:0) (Figure 8).

### 3. Epimastigotes are able to disassemble the lipid inclusions inside reservosomes

**Figure 9:**
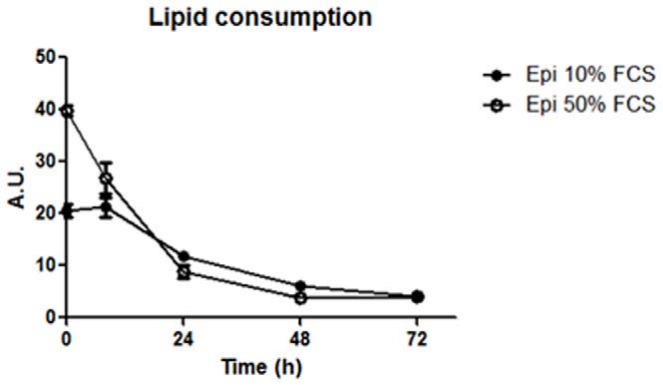
Lipid consumption of epimastigotes supplemented with 10 or 50% FCS. Cells were placed in serum-free medium for 0, 8, 24, 48 and 72 h. Note that in both initial culture conditions, the fluorescence decreases along time in parasites from 10% FCS or 50% FCS. Results of two independent experiments, performed in triplicate. The data was analyzed with two - way ANOVA test and post analyzed by Bonferroni test (P<0.05). A.U., arbitrary units.

**Figure 10:**
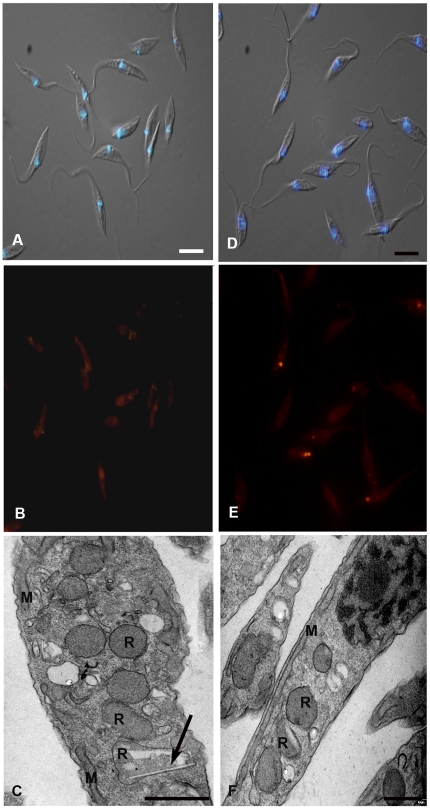
The neutral lipid stock inside epimastigotes decreases along time in serum-free medium. The Figure shows the absence of fluorescence almost complete in epimastigotes from 10 (B) and 50% FCS (E) after 48 h of serum starvation. Ultrathin sections revealed that the majority of reservosomes are devoid of lipid inclusions, while others still present rectangular lipid profiles in 10 (C) and 50% (F). Merged images of DIC and DAPI fluorescence were used to point nucleus and kinetoplast position (A, C). The asterisks indicate the lipid inclusions. Bars: 10 µm (a–B, D–E) and 0.5 µm (C–F).

Epimastigotes originated from 10 and 50% FCS cultures were maintained in LIT medium without serum for 0, 8, 24, 48 and 72 hours. We could follow lipid consumption by fluorimetric analysis of Nile Red fluorescence (Figure 9) and observe the reservosomes by electron microscopy. Epimastigote lipid content decreased 32.5% in parasites grown in 50% FCS after 8 h of serum starvation, whereas no significant difference was found in epimastigotes cultivated in 10% FCS after the same period. After 24 h, the reduction was 42.4% and 77.9% in parasites grown in 10 and 50% FCS, respectively. Interestingly, reservosome neutral lipid consumption reaches, approximately, 80% and 90% in 10%- and 50%- FCS culture, respectively, after 48 h (Figure 9). Fluorescence images show few reservosomes positively stained with Nile Red, probably due to the low amount of lipids in their lumen (Figures 10 B and E). Using electron microscopy, we could find some lipid-devoid reservosomes co-existing with organelles displaying few lipid inclusions (Figures 10C and F). Together, these results suggest the ability of epimastigotes to mobilize neutral lipid reservoir in reservosomes according to cellular demand. In all experiments, the percentile of cell death using Sytox Blue did not overcome 13%. We have analysed the area occupied by lipid inclusions in epimastigotes cultivated in LIT supplemented with 10% complete FCS serum (control parasites) before and after incubation in LIT with 10% delipidated FCS. Morphometric analyses (Table 1) showed a reduction of 70% in lipid inclusions.

**Table 1:** Morphometric comparison of the area occupied by lipid inclusions in epimastigotes cultivated in control conditions (time zero) or after 48 h of incubation in LIT supplemented with 10% dFCS.

Parasites (epimastigotes)	Number of parasites analyzed	Number of reservosomes analyzed	Mean area of lipid inclusions per reservosome (µm^2^)	Percentage of lipid inclusion reduction
Time Zero	50	103	0.05607±0.003011	-
48 h in dFCS	50	122	0.01677±0.002546	70.21%*

*One tailed *t* test, P<0.0001.

## Discussion

*T. cruzi* depends on exogenous lipids from host to play essential roles in all developmental forms, as growth and host infection. Changes in phospholipid and neutral lipid profiles resulting from aging in culture or temperature shift of epimastigotes or trypomastigotes were already reported [34], [35]. Amastigotes contain high amounts of cholesterol, probably derived from host cells [36] and a complete absence of ergosterol, the major neutral lipid present in epimastigotes from reduviid vector or grown in axenic LIT medium [34], [37], [38]. LDL-endocytosis and accumulation in reservosomes have only been documented in the epimastigote form [14]. These works have shown the ability of the parasites to modulate their lipid content according to the medium and cell requirements. However, very little information about lipid traffic in *T. cruzi* is available. In this way, reservosomes, the final step in the epimastigote endocytic pathway, exercise a pivotal role in storing and disposing lipids and proteins uptaken by endocytosis [20], [ 39], [ reviewed in 16].

Since trypanosomatids do not synthesize cholesterol (or cholesteryl ester derivatives) [reviewed in 40] and as cholesterol acquisition in epimastigotes has never been quantified, we decided to analyze the reservosome neutral lipid content in response to serum concentration in culture medium. Although epimastigotes are able to proliferate in low-serum (1% FCS) concentrations, lipid reservoir in these parasites diminished in comparison to control (10% FCS), their reservosomes are devoid of lipid inclusions, and some of them seem to fuse with each other (Fig. 4 A–B). Homotypic fusion events suggest the reduction in reservosome number. On the other hand, parasites cultivated in 50% FCS-supplemented medium store over twice as much neutral lipids than parasites grown in 10% FCS. Surprisingly, cell proliferation was higher in epimastigotes from 10%-FCS than 50%-FCS medium. The yellow-gold fluorescence concentrated in internal compartments of these parasites (Fig. 3F) suggested that reservosomes and possibly cytoplasmic lipid bodies were the lipid storage site. Nile red fluorimetry of isolated reservosomes from epimastigotes grown in 50% FCS (Fig. 5) and electron microscopy (Fig. 4 E–G), confirmed this hypothesis. The biochemical composition of reservosome lipid inclusions was further determined by GC-MS and confirmed cholesterol and cholesteryl esters as the major neutral lipids (Fig. 8). In higher eukaryotes, cholesterol reaches the internal milieu by LDL receptor-mediated endocytosis. In *T. cruzi*, the LDL receptor was not characterized, although LDL endocytosis and accumulation in reservosomes have already been demonstrated [14]. The bloodstream and procyclic forms of *T. brucei* require lipoproteins (LDL and HDL) and other serum components to growth and maintenance in axenic cultures, providing cholesterol, cholesterol esters and phospholipids to the parasites [41], [42]. Many works characterized LDL or HDL receptors in *T. brucei* and in other trypanosomatids such as *T. b. rhodesiense*, *Leishmania donovani* and *Crithidia luciliae* [43], [44], [45]. In this way, the sterol acquisition by *T. cruzi* epimastigote could operate by similar mechanisms.

In our current analysis, ergosterol represented only 1.5% of neutral lipids in reservosomes, being completely absent in the lipid inclusion fraction. This clarifies the real nature of reservosome lipid inclusions, and highlights the role of cholesterol from serum in the formation of lipid rods. The low concentration of ergosterol in reservosomes is in agreement with early work concerning ergosterol distribution in epimastigote subcellular fractions [46]: they found ergosterol associated with mitochondria and microsomes. We had found higher ergosterol content (22% of neutral lipids) in the preliminary lipid analysis of reservosome fraction [24], probably due to some contamination with mitochondria, since we also identified the presence of a small content of cardiolipin, a mitochondrion marker phospholipid. From 2002 to now, we have improved our fractionation protocol, choosing to work with another band (B1 instead of B2) of the reservosome purification gradient that gives higher organelle enrichment, although with lower yield [19]. Mitochondria are also the most affected organelle in cells incubated in the presence of ergosterol synthesis inhibitors [13]. Another important indication of the coherence of our data comes from the careful examination of the electron micrographs published by Lazardi and coworkers in 1990 [47, Figures 2A–C and 3A therein] that show the ultrastructural alterations caused by the treatment of epimastigotes with ketoconazol and terbinafine, potent ergosterol biosynthesis inhibitors. Although the authors did not mention at that time, it is possible to observe that reservosome lipid inclusions were not affected.

In this work we have obtained the first fatty acid profile of reservosomes. Like other lipids, fatty acid biosynthesis is constantly submitted to environmental conditions, which includes temperature, availability in the culture medium and aging [48]. Indeed, epimastigotes have C18:1 (oleate) and C18:2 (linoleate) in high quantities [33], [49]. In our analysis, fatty acid composition in reservosomes is probably a result of lipid endocytosis and parasite production, since the organelle is formed by fusion of Golgi vesicles with endocytic vesicles from plasma membrane [50].

Taken together, these results support the idea that the neutral lipid content of reservosomes is a consequence of intense acquisition of exogenous lipids. Reservosomes containing sword-shaped lipid inclusion profiles surrounded by a phospholipid monolayer have already been shown by our group [21]. In mammals, the high acquisition rate of cholesterol-loaded LDL and its accumulation in lysosomes, where events of crystallization can occur [51], [52], contributes to the formation of foam cells and development of atherosclerosis in arterial intima. Like late endosomes and lysosomes, reservosomes are acidic organelles and may render favorable milieu to crystal formation. However, unlike human foam cell lysosomes, reservosomes may be capable of disassemble cholesterol crystalloid inclusions. Previous work had shown a progressive involution of reservosomes during metacyclogenesis [39], a period of parasite life cycle when the demand for energy is high and external substrate supply is very low. Now we have submitted epimastigotes harboring cholesterol-loaded reservosomes to conditions of low cholesterol supply (serum-free medium), but not sufficiently low to trigger metacyclogenesis. After 48 h in this condition, 90% of epimastigote neutral lipids had been consumed (Fig. 9). Parasites were alive and their reservosomes did not present crystalloid lipid inclusions anymore, although some rectangular inclusions could be rarely found (Fig. 10). The area occupied by inclusions inside reservomes was reduced in 70% (Table 1). These results are in agreement with the concept that the major role of reservosomes is nutrient storage to be consumed according to cellular demand [39], [53]. In our experiments we did not observe metacyclic trypomastigote forms as a result of serum starvation, which could be due to the rich composition of the LIT medium.

The mechanisms used by *T. cruzi* epimastigotes to mobilize reservosome lipid inclusions remain largely unknown. The reservosome proteomic analysis failed to identify proteins usually related to the efflux and cellular distribution of cholesterol, like NPC1 and NPC2. However, other proteins were found that could be involved in lipid transport from reservosomes into cytoplasm, as an ABCA1 transporter and Rab18 [19], [54]. The cellular function of ABCA1 is correlated to its localization in plasma membrane, aiding in cholesterol efflux [52], notably in macrophages. The presence of ABCA1 in endocytic compartments has already been reported by Neufeld and colleagues [55], [56]. Moreover, the group demonstrated that the ABCA1 transporter acts in lipid efflux from late endosomes. In *T. cruzi*, ABCA1 was localized at the plasma membrane, flagellar pocket and endocytic compartments. It could function in extruding cholesterol excess back to extracellular milieu, as it does in mammals. The presence of ABCA1 in endocytic compartments may be correlated to its own turnover or, alternatively, to the extrusion of cholesterol from reservosomes to the cytosol. Rab18 is commonly found in lipid bodies and endoplasmic reticulum of many cell types, where it is involved in releasing lipids from these internal reservoirs [12], [57]. The presence of a Rab 18 homologue in *T. cruzi* reservosomes [19] may also be correlated to lipid distribution to other organelles. Additional studies are been performed to address this hypothesis.

## Supporting Information

Figure S1 **Thin layer chromatography from medium and parasites in different culture conditions.** A – Liver Infusion Triptose (LIT) Medium; B – LIT with 10% Fetal Calf Serum (FCS); C – LIT with 10% delipidated Fetal Calf Serum; D – 10% Fetal Calf Serum; E – 10% delipidated Fetal Calf Serum (dFCS); F – *T.cruzi* Epimastigotes in LIT+10% FCS (1×10^8^ cells); G - *T.cruzi* Epimastigotes in LIT+10% dFCS for 48 h (1×10^8^ cells); H - *T.cruzi* Epimastigotes in LIT+10% dFCS+1 mg/mL human LDL (1×10^8^ cells); I - *T.cruzi* Epimastigotes in LIT+10% dFCS+2 mg/mL human LDL (1×10^8^ cells). Lipids: HC – Hydrocarbon; CHOE – Cholesteryl-ester; ERGE – Ergosteryl-ester; TG – Triacylglycerol; FA – Free Fatty Acids; CHO – Cholesterol; ERG – Ergosterol; MG – Monoacylglycerol; PL – Phospholipids; ND – Not Determined.
(JPG)

Figure S2 **Fluorimetric analysis using Nile Red of epimastigotes in different culture conditions.** Control epimastigotes, cultivated in 10% FCS, store twice as much neutral lipids than those grown in 10% delipidated FCS. After incubation with purified 1 mg/mL LDL the parasites presented 6 times more neutral lipids and after incubation with 2 mg/mL LDL, the store amount reaches 8 times the capacity of control epimastigotes. Fluorescence intensity was expressed in arbitrary units. The results are from two independent experiments.
(PPT)

## References

[1] Hao M, Lin SX, Karylowski OJ, Wüstner D, McGraw TE (2002). Vesicular and non-vesicular sterol transport in living cells. The endocytic recycling compartment is a major sterol storage organelle.. J Biol Chem.

[2] Mobius W, van Donselaar E, Ohno-Iwashita Y, Shimada Y, Heijnen HF (2003). Recycling compartments and the internal vesicles of multivesicular bodies harbor most of the cholesterol found in the endocytic pathway.. Traffic.

[3] Holtta-Vuori MA, Uronen RL, Repakova J, Salonen E, Vattulainen I (2008). BODIPY-cholesterol: a new tool to visualize sterol trafficking in living cells and organisms.. Traffic.

[4] Lingwood D, Simons K (2010). Lipid rafts as a membrane-organizing principle.. Science.

[5] Ikonen E (2006). Mechanisms for cellular cholesterol transport: defects and human disease.. Physiol Rev.

[6] Devlin C, Pipalia NH, Liao X, Schuchman EH, Maxfield FR, Tabas I (2010). Improvement in lipid and protein trafficking in NPC1 cells by correction of a secondary enzyme defect.. Traffic.

[7] Goldstein JL, Brown MS (2009). The LDL receptor.. Arterioscler Thromb Vasc Biol.

[8] Soccio RE, Breslow JL (2004). Intracellular Cholesterol Transport.. Arterioscler Thromb Vasc Biol.

[9] Ikonen E (2008). Cellular cholesterol trafficking and compartmentalization.. Nat Rev Mol Cell Biol.

[10] Sugii S, Reid PC, Ohgami N, Du H, Chang TY (2003). Distinct endosomal compartments in early trafficking of low density lipoprotein-derived cholesterol.. J Biol Chem.

[11] Mesmin B, Maxfield FR (2009). Intracellular sterol dynamics.. Biochim Biophys Acta.

[12] Martin S, Parton RG (2006). Lipid droplets: a unified view of dynamic organelle.. Nature Rev Mol Cell Biol.

[13] Urbina JA (2009). Ergosterol biosynthesis and drug development for Chagas disease.. Mem Inst Oswaldo Cruz.

[14] Soares MJ, De Souza W (1991). Endocytosis of gold-labeled proteins and LDL by *Trypanosoma cruzi*.. Parasitol Res.

[15] Porto Carreiro IA, Miranda K, Attias M, De Souza W, Cunha-e-Silva NL (2000). *Trypanosoma cruzi* epimastigotes endocytic pathway: cargo enters the cytostome and passes through an early endosomal network before reservome storage.. Eur J Cell Biol.

[16] De Souza W, Sant'Anna C, Cunha-e-Silva NL (2009). Electron microscopy and cytochemistry analysis of the endocytic pathway of pathogenic protozoa.. Progress Histochem Cytochem.

[17] Sant'Anna C, Parussini F, Lourenço D, De Souza W, Cazzulo JJ (2008). All *Trypanosoma cruzi* developmental forms present lysosome-related organelles.. Histochem Cell Biol.

[18] Cunha-e-Silva N, Sant'anna C, Pereira MG, Porto-Carreiro I, Jeovanio AL (2006). Reservosomes: multipurpose organelles?. Parasitol Res.

[19] Sant'Anna C, Nakayasu ES, Pereira MG, Lourenço D, De Souza W (2009). Subcellular proteomics of *Trypanosoma cruzi* reservosomes.. Proteomics.

[20] Soares MJ, De Souza W (1988). Cytoplasmic organelles of trypanosomatids. A cytochemical and stereological study.. J Submicrosc Cytol Pathol.

[21] Sant'Anna C, Pereira MG, Lemgruber L, de Souza W, Cunha-e-Silva NL (2008). New insights into the morphology of *Trypanosoma cruzi* reservosome.. Microsc Res Tech.

[22] Tangirala RK, Jerome WG, Jones NL, Small DM, Johnson WJ (1994). Formation of cholesterol monohydrate crystals in macrophage-derived foam cells.. J Lipid Res.

[23] Klinkner AM, Waites CR, Kerns WD, Bugelski PJ (1995). Evidence of foam cell and cholesterol crystal formation in macrophages incubated with oxidized LDL by fluorescence and electron microscopy.. J Histochem Cytochem.

[24] Cunha-e-Silva NL, Atella GC, Porto-Carreiro IA, Morgado-Diaz JA, Pereira MG (2002). Isolation and characterization of a reservosome fraction from *Trypanosoma cruzi*.. FEMS Microbiol Lett.

[25] Camargo EP (1964). Growth and differentiation of *Trypanosoma cruzi*. I. Origin of metacyclic trypanosomes in liquid media.. Rev Inst Med Trop São Paulo.

[26] Chapman MJ, Goldstein S, Lagrange D, Laplaud PM (1981). A density gradient ultracentrifugal procedure for the isolation of the major lipoprotein classes from human serum.. J Lipid Res.

[27] Cham BE, Knowles BR (1976). A solvent system for delipidation of plasma or serum without protein precipitation.. Journal of Lipid Research.

[28] Liu P, Ying Y, Zhao Y, Mundy DJ, Zhu M (2004). Chinese hamster ovary K2 cell lipid droplets appear to be metabolic organelles involved in membrane traffic.. J Biol Chem.

[29] Bligh EG, Dyer WJ (1959). A rapid method for total lipid extraction and purification.. Can J Biochem Physiol.

[30] Almeida IC, Camargo MM, Procópio DO, Silva LS, Mehlert A (2000). Highly purified glycosylphosphatidylinositols from *Trypanosoma cruzi* are potent proinflammatory agents.. EMBO J.

[31] Pernet F, Pelletier CJ, Milley J (2006). Comparison of three solid-phase extraction methods for fatty acid analysis of lipid fractions in tissues of marine bivalves.. J Chromatogr A.

[32] Fridberg A, Olson CL, Nakayasu ES, Tyler KM, Almeida IC (2008). Sphingolipid synthesis is necessary for kinetoplast segregation and cytokinesis in *Trypanosoma brucei*.. J Cell Sci.

[33] Maldonado RA, Kuniyoshi RK, Linss JG, Almeida IC (2006). *Trypanosoma cruzi* oleate desaturase: molecular characterization and comparative analysis in other trypanosomatids.. J Parasitol.

[34] Bronia DH, Aguerri AM, Bertetto ST (1986). *Trypanosoma cruzi*: changes in lipid composition during aging in culture.. Exp Parasitol.

[35] Florin-Christensen M, Florin-Christensen J, de Isola ED, Lammel E, Meinardi E (1997). Temperature acclimation of *Trypanosoma cruzi* epimastigote and metacyclic trypomastigote lipids.. Mol Biochem Parasitol.

[36] Liendo A, Visbal G, Piras MM, Piras R, Urbina JA (1999). Sterol composition and biosynthesis in *Trypanosoma cruzi* amastigotes.. Mol Biochem Parasitol.

[37] Isola EL, Lammel EM, González Cappa SM (1986). *Trypanosoma cruzi*: differentiation after interaction of epimastigotes and *Triatoma infestans* intestinal homogenate.. Exp Parasitol.

[38] Urbina JA, Lazardi K, Marchan E, Visbal G, Aguirre T (1993). Mevinolin (lovastatin) potentiates the antiproliferative effects of ketoconazole and terbinafine against *Trypanosoma (Schizotrypanum) cruzi*: in vitro and in vivo studies.. Antimicrob Agents Chemother.

[39] Soares MJ, Souto-Padron T, Bonaldo MC, Goldenberg S, De Souza W (1989). A stereological study of the differentiation process in *Trypanosoma cruzi*.. Parasitol Res.

[40] De Souza W, Rodrigues JCF (2009). Sterol biosynthesis pathway as target for anti-trypanosomatid drugs.. Interdiscipl Perspect Infect Dis.

[41] Coppens I, Baudhuin P, Opperdoes FR, Courtoy PJ (1988). Receptors for the host low density lipoproteins on the hemoflagellate *Trypanosoma brucei*: purification and involvement in the growth of the parasite.. Proc Natl Acad Sci USA.

[42] Lee MG, Yen FT, Zhang Y, Bihain BE (1999). Acquisition of lipoproteins in the procyclic form of *Trypanosoma brucei*.. Mol Biochem Parasitol.

[43] Bastin P, Stephan A, Raper J, Saint-Remy JM, Opperdoes FR (1996). An M(r) 145,000 low-density lipoprotein (LDL)-binding protein is conserved throughout the Kinetoplastida order.. Mol Biochem Parasitol.

[44] Liu J, Qiao X, Du D, Lee MG (2000). Receptor-mediated endocytosis in the procyclic form of *Trypanosoma brucei*.. J Biol Chem.

[45] Green HP, Del Pilar Molina Portela M, St Jean EN, Lugli EB (2003). Evidence for a *Trypanosoma brucei* lipoprotein scavenger receptor.. J Biol Chem.

[46] Turrens JF, Boveris A, Gros EG, Stoppani AOM (1980). Distribucion subcelular de ergosterol y esteroles 5,7-dienicos en *Trypanosoma cruzi*.. Medicina.

[47] Lazardi K, Urbina JA, De Souza W (1990). Ultrastructural alterations induced by two ergosterol biosynthesis inhibitors, ketoconazole and terbinafine, on epimastigotes and amastigotes of *Trypanosoma (Schizotrypanum) cruzi*.. Antimicrob Agent Chemoth.

[48] Villasuso AL, Aveldaño M, Vicario A, Machado-Domenech EE, Garcia de Lema M (2005). Culture age and carbamoylcholine increase the incorporation of endogenously synthesized linoleic acid in lipids of *Trypanosoma cruzi* epimastigotes.. Biochim Biophys Acta.

[49] Esteves MG, Gonzales-Perdomo M, Alviano CS, Angluster J, Goldenberg S (1989). Changes in fatty acid composition associated with differentiation of *Trypanosoma cruzi*.. FEMS Microbiol Lett.

[50] Sant'Anna C, De Souza W, Cunha-e-Silva N (2004). Biogenesis of the reservosomes of *Trypanosoma cruzi*.. Microsc Microanal.

[51] Bobryshev Y (2006). Monocyte recruitment and foam cell formation in atherosclerosis.. Micron.

[52] Cavelier C, Lorenzi I, Rohrer L, Von Eckardstein A (2006). Lipid efflux by the ATP-binding cassette transporters ABCA1 and ABCG1.. Biochim Biophys Acta.

[53] Figueiredo RC, Rosa DS, Soares MJ (2000). Differentiation of *Trypanosoma cruzi* epimastigotes: metacyclogenesis and adhesion to substrate are triggered by nutritional stress.. J Parasitol.

[54] Torres C, Perez-Victoria FJ, Parodi-Talice A, Castanys S, Gamarro F (2004). Characterization of an ABCA-like transporter involved in vesicular trafficking in the protozoan parasite *Trypanosoma cruzi*.. Mol Microbiol.

[55] Neufeld EB, Remaley AT, Demosky SJ, Stonik JA, Cooney AM (2001). Cellular localization and trafficking of the human ABCA1 transporter.. J Biol Chem.

[56] Neufeld EB, Stonik JA, Demosky SJ, Knapper CL, Combs CA (2004). The ABCA1 transporter modulates late endocytic trafficking: insights from the correction of the genetic defect in Tangier disease.. J Biol Chem.

[57] Martin S, Parton RG (2008). Characterization of Rab18, a lipid droplet-associated small GTPase.. Methods Enzymol.

