# Supplementary figures and images for: Trypanosoma cruzi Epimastigotes Are Able to Store and Mobilize High Amounts of Cholesterol in Reservosome Lipid Inclusions
Source: PLoS One. 2011 Jul 27;6(7):e22359. doi: 10.1371/journal.pone.0022359 (PMC3144899; doi:10.1371/journal.pone.0022359)

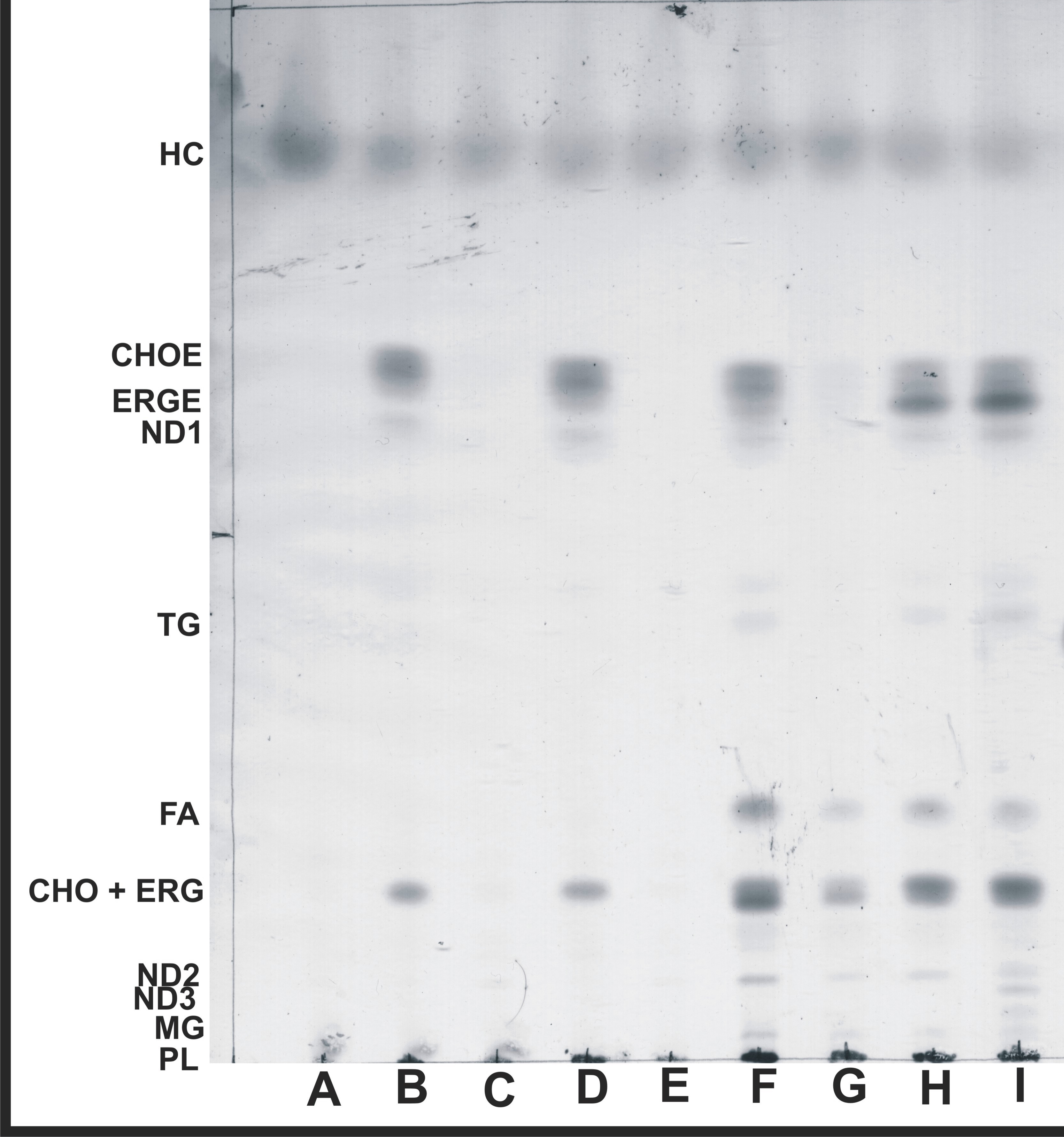

Supplement: Figure S1 — Thin layer chromatography from medium and parasites in different culture conditions. A – Liver Infusion Triptose (LIT) Medium; B – LIT with 10% Fetal Calf Serum (FCS); C – LIT with 10% delipidated Fetal Calf Serum; D – 10% Fetal Calf Serum; E – 10% delipidated Fetal Calf Serum (dFCS); F – T.cruzi Epimastigotes in LIT+10% FCS (1×10^8^ cells); G - T.cruzi Epimastigotes in LIT+10% dFCS for 48 h (1×10^8^ cells); H - T.cruzi Epimastigotes in LIT+10% dFCS+1 mg/mL human LDL (1×10^8^ cells); I - T.cruzi Epimastigotes in LIT+10% dFCS+2 mg/mL human LDL (1×10^8^ cells). Lipids: HC – Hydrocarbon; CHOE – Cholesteryl-ester; ERGE – Ergosteryl-ester; TG – Triacylglycerol; FA – Free Fatty Acids; CHO – Cholesterol; ERG – Ergosterol; MG – Monoacylglycerol; PL – Phospholipids; ND – Not Determined. (JPG) [file pone.0022359.s001.jpg]

## Slide 1
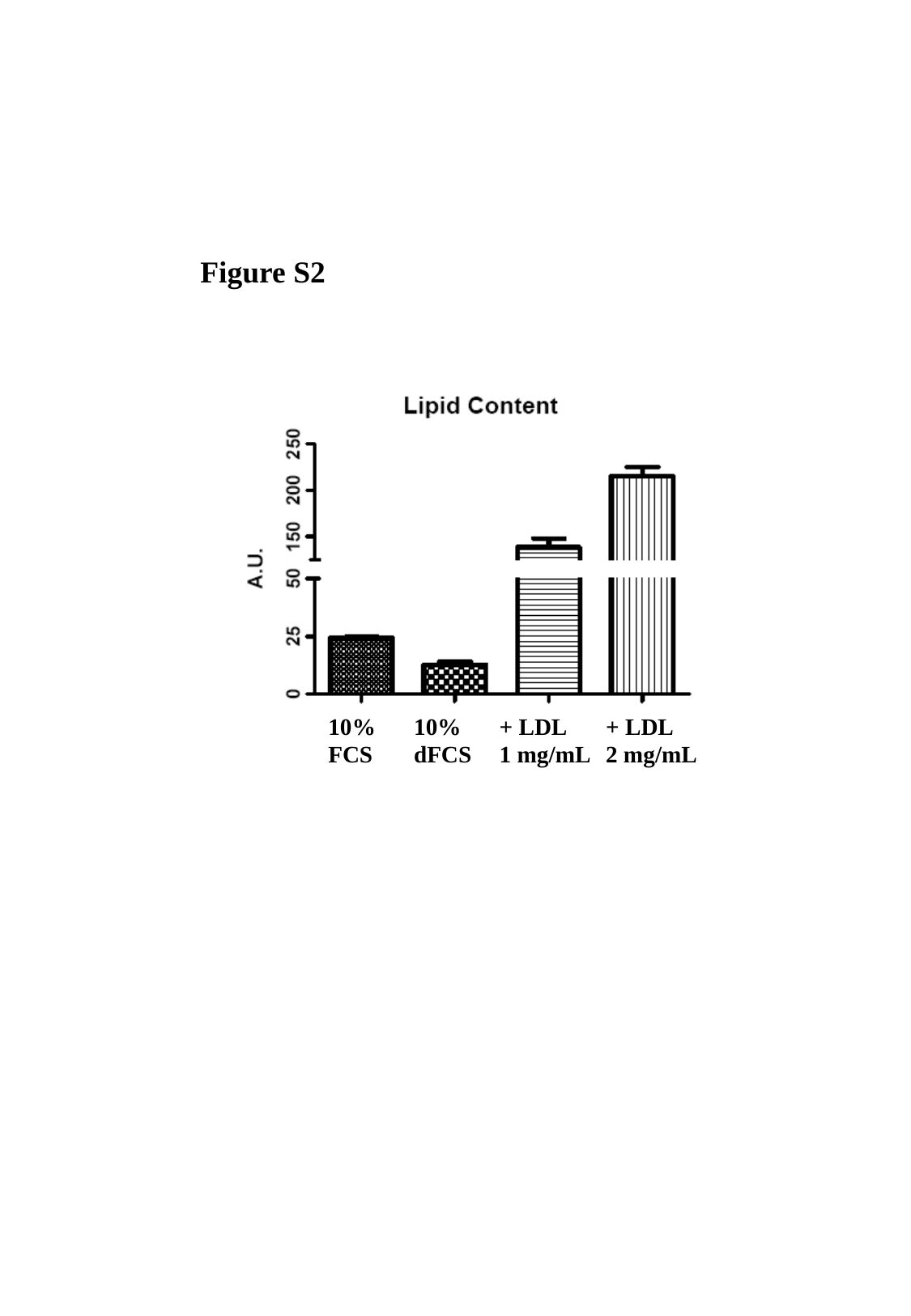

Figure S2
10%
FCS
10%
dFCS
+ LDL
1 mg/mL
+ LDL
2 mg/mL

Supplement: Figure S2 — Fluorimetric analysis using Nile Red of epimastigotes in different culture conditions. Control epimastigotes, cultivated in 10% FCS, store twice as much neutral lipids than those grown in 10% delipidated FCS. After incubation with purified 1 mg/mL LDL the parasites presented 6 times more neutral lipids and after incubation with 2 mg/mL LDL, the store amount reaches 8 times the capacity of control epimastigotes. Fluorescence intensity was expressed in arbitrary units. The results are from two independent experiments. (PPT) [file pone.0022359.s002.ppt]
